# Supplementary material for: Comparison of Phenolic Compounds and Evaluation of Antioxidant Properties of Porophyllum ruderale (Jacq.) Cass (Asteraceae) from Different Geographical Areas of Queretaro (Mexico)
Source: Plants (Basel). 2023 Oct 14;12(20):3569. doi: 10.3390/plants12203569 (PMC10609970; doi:10.3390/plants12203569)
Supplement: Supplementary file 1 [file plants-12-03569-s001.zip › plants-2643979-supplementary.pdf]

plants

MDPI

Supplementary Information

Comparison of Phenolic Compounds and Evaluation of Antioxidant Properties of *Porophyllum ruderale* (Jacq.) Cass (*Asteraceae*) from Different Geographical Areas of Queretaro (Mexico)

Ángel Félix Vargas-Madriz [1], Ivan Luzardo-Ocampo [2,3], Jorge Luis Chávez-Servín [1], Ulisses Moreno-Celis [1], Octavio Roldán-Padrón [1], Haidel Vargas-Madriz [4], Haydé Azeneth Vergara-Castañeda [5] and Aarón Kuri-García [1,*]

[1] Department of Cell and Molecular Biology, School of Natural Sciences, Universidad Autonoma de Queretaro, Qro 76230, Mexico; angel.vargas@uaq.mx (Á.F.V.-M.); jorge.chavez@uaq.mx (J.L.C.-S.); ulisses.moreno@uaq.mx (U.M.-C.); octavio.roldan@uaq.mx (O.R.-P.)
[2] Tecnologico de Monterrey, The Institute for Obesity Research, Ave. Eugenio Garza Sada 2501 Sur, Monterrey 64841, Mexico; ivan.8907@gmail.com
[3] Tecnologico de Monterrey, School of Engineering and Sciences, Campus Guadalajara, Av. General Ramon Corona 2514, Zapopan 45201, Mexico
[4] Department of Agricultural Production, Centro Universitario de la Costa Sur, University of Guadalajara, Av. Independencia Nacional, No 151, Autlán 48900, Mexico; haidel_vargas@hotmail.com
[5] Advanced Biomedical Research Center, School of Medicine, Universidad Autonoma de Queretaro, Qro 76140, Mexico; hayde.vergara@uaq.mx
* Correspondence: aaron.kuri@uaq.mx

Citation: Vargas-Madriz, Á.F.; Luzardo-Ocampo, I.; Chávez-Servín, J.L.; Moreno-Celis, U.; Roldán-Padrón, O.; Vargas-Madriz, H.; Vergara-Castañeda, H.A.; Kuri-García, A. Comparison of Phenolic Compounds and Evaluation of Antioxidant Properties of *Porophyllum ruderale* (Jacq.) Cass (*Asteraceae*) from Different Geographical Areas of Queretaro (Mexico). *Plants* **2023**, *12*, 3569. https://doi.org/10.3390/plants12203569

Academic Editor: Ain Raal

Received: 18 September 2023
Revised: 10 October 2023
Accepted: 11 October 2023
Published: 14 October 2023

Copyright: © 2023 by the authors. Licensee MDPI, Basel, Switzerland. This article is an open access article distributed under the terms and conditions of the Creative Commons Attribution (CC BY) license (https://creativecommons.org/licenses/by/4.0/).

Supplementary Table S1. Content of phenolic compounds quantified by HPLC-DAD for *P. ruderale* from Landa de Matamoros and Jalpan de Serra.

Compound name	RT (min)	Landa de Matamoros (mg eq./g DW)	Arroyo Seco (mg eq./g DW)
Hydroxycinnamic acids and derivatives			
Chlorogenic acid	10.58	12.3 ± 0.2	8.5 ± 0.04*
Sinapic acid	11.67	5.3 ± 0.1	6.5 ± 0.07*
Caffeic acid	11.82	6.7 ± 0.1	0.6 ± 0.03*
p-Coumaric acid	14.42	18.8 ± 0.1	4.5 ± 0.01*
Ferulic acid	15.35	19.4 ± 0.3	2.1 ± 0.02*
Hydroxybenzoic acids and derivatives and benzaldehydes			
Gallic acid	7.49	1.2 ± 0.01	2.6 ± 0.01*
Hydroxybenzoic acid	12.35	11.3 ± 0.01	2.3 ± 0.02*
Benzenoids			
Hydroxyphenylacetic acid	9.89	20.3 ± 0.01	6.7 ± 0.03*
Flavonols			
Rutin	12.9	1.2 ± 0.01	2.0 ± 0.17*
Quercetin	18.44	0.5 ± 0.01	0.1 ± 0.01*
(+)-catechin	10.85	73.4 ± 0.4	106.4 ± 0.76*
Epicatechin	11.55	240.7 ± 2.0	195.2 ± 4.02*
Epigallocatechin gallate	11.8	36.1 ± 0.01	39.7 ± 0.14*

The results are expressed as the mean ± S.D. of three independent extractions, in triplicate. The asterisks indicate significant differences ($p<0.05$) by Student's test. DW: dry weight; RT: retention time.

Supplementary Table S2. Principal components, percentual participation, and cumulative percent.

Principal component (PC)	Percent	Cumulative percent
1	92.75	92.75
2	5.03	97.78
3	2.22	100.00

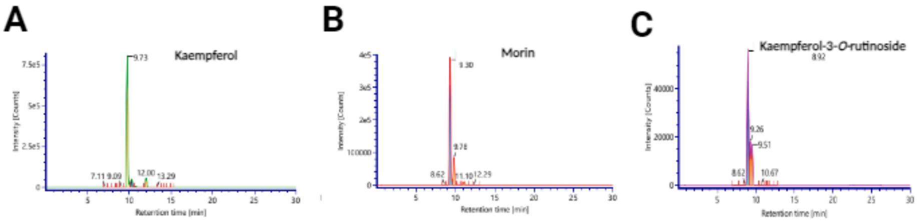

Supplementary Figure S1. Representative pictures from the Mass spectrometry analysis (ESI+) of some of the identified metabolites in *P. ruderale* leaves. **(A)** Kaempferol, **(B)** Morin, **(C)** Kaempferol-3- *O*-rutinoside

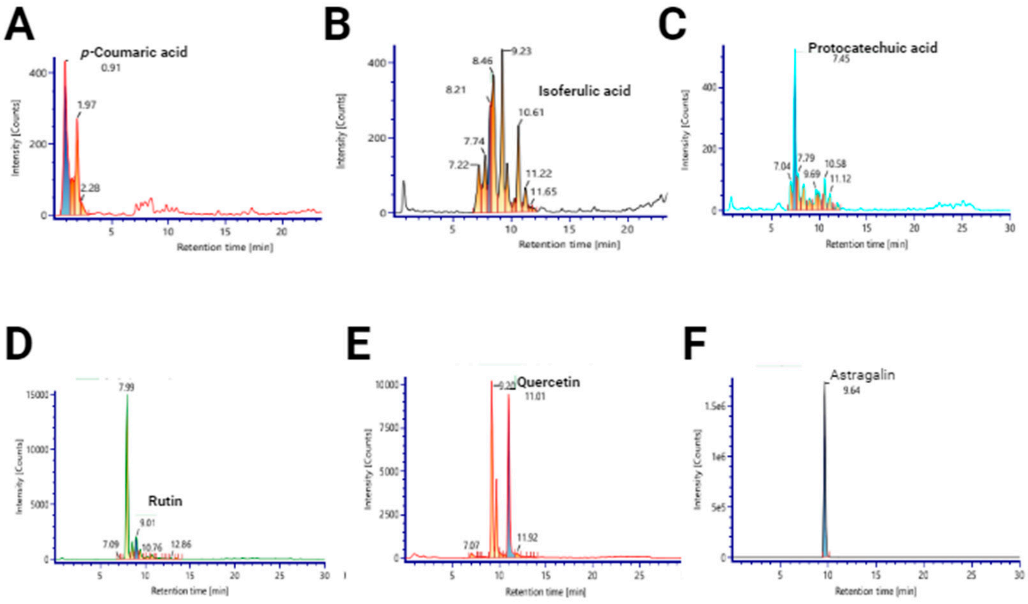

Supplementary Figure S2. Representative pictures from the Mass spectrometry analysis (ESI-) of some of the identified metabolites in *P. ruderale* leaves. **(A)** *p*-Cumaric acid, **(B)** Isoferulic acid, **(C)** Protocatechuic acid, **(D)** Rutin, **(E)** Quercetin, **(F)** Astragalin.

Supplementary Table S3. Curves for each standard used in the HPLC-DAD analysis.

Compound	Standard Curve	R^2
Chlorogenic acid	y=18434x + 90.485	0.991
Sinapic acid	y=25013x – 3.5893	0.999
Caffeic acid	y=42480x – 19.435	0.999
p-Coumaric acid	y=803.24x + 71.788	1.000
Ferulic acid	y=38663x + 1851.8	0.996
Gallic acid	y=26099x + 275.90	0.999
Hydroxybenzoic acid	y=4.2007x – 78.089	0.990
Hydroxyphenylacetic acid	y=3.4788x – 187.63	0.998
Rutin	y=20150x + 222.76	1.000
Quercetin	y=50105x – 397.9	0.995
(+)-catechin	y=7835.1x+20.686	0.997
Epicatechin	y=10.072x – 143.04	0.991
Epicatechin gallate	y=3.7738x – 26.299	0.990
